# Effects of fermented jujube powder on growth performance, rumen fermentation, and antioxidant properties of simmental bulls

**DOI:** 10.3389/fvets.2024.1442244

**Published:** 2024-07-31

**Authors:** Yongqing Liu, Gaifang Wang, Rui Wang, Xia Zhang, Caiping Feng, Ying He, Panpan Chu

**Affiliations:** Department of Biology and Food Engineering, Lyuliang University, Lüliang, China

**Keywords:** fermented jujube powder, production performance, rumen fermentation, antioxidant capacity, bulls

## Abstract

**Introduction:**

Fermented jujube powder (FJP) promotes a balance between the intestinal microflora and immune factors in animals. In this study, we aimed to investigate the effects of FJP on the production performance, nutrient digestion, rumen fermentation, and antioxidant properties of bulls.

**Methods:**

Forty Simmental bulls were randomly divided into four groups based on body weight and fed a basal diet with [5, 7.5, or 10% dry matter (DM)] or without FJP. The experimental period was 20 d for adaptation and 60 d for the feeding trial.

**Results:**

Dietary FJP supplementation did not affect DM intake (*P* > 0.05) but increased the average daily gain quadratically (*P* = 0.049) and decreased the feed conversion ratio linearly (*P* = 0.042). FJP quadratically enhanced DM and crude protein digestibility (*P* = 0.026 and *P* = 0.041, respectively) and linearly enhanced acid detergent fiber digestibility (*P* = 0.048). It also increased the total volatile fatty acid concentration quadratically (*P* = 0.037), acetate molar percentage, and acetate-to-propionate ratio linearly (*P* = 0.002 and 0.001), and reduced the ammonia nitrogen concentration linearly (*P* = 0.003). Additionally, xylanase and protease activities and *Ruminococcus flavefaciens* abundance increased linearly (*P* = 0.006, 0.018, and 0.009, respectively), and total bacteria, *Ruminococcus albus*, and *Ruminobacter amylophilus* abundance increased quadratically (*P* = 0.047, 0.011, and 0.021, respectively). FJP linearly increased serum total protein concentration and antioxidant capacity (*P* = 0.003 and 0.018, respectively) and decreased malonaldehyde content (*P* = 0.006).

**Discussion:**

FJP supplementation (7.5%) enhanced production performance, nutrient digestion, rumen fermentation, and serum antioxidant capacity in bulls. The improved nutrient digestion may be due to an increase in ruminal microorganisms and total volatile fatty acids from the FJP. High blood antioxidant levels indicate that FJP may preserve proteins, thereby boosting the production performance of bulls.

## 1 Introduction

Rising animal feed prices necessitate the inclusion of less expensive regionally produced ingredients as unconventional feedstocks (1–5). Jujube (*Ziziphus jujube*), also known as Chinese date, holds significant importance in nutritional, medicinal, industrial, and cultural domains (6). Jujube fruits are used in various food products, and industrial processing of jujube generates large amounts of by-products, which are rich sources of nutrients and exhibits potential health benefit (7). Therefore, consideration of jujube by-products as a feed source has gained increased interest. China is the leading producer of jujube fruit worldwide and produced 7.46 million tons in 2019; however, an estimated 1–1.5 million tons were not suitable for consumption (7). Therefore, using jujube as a feed source not only reduces waste but also alleviates feed resource shortages. Jujube is rich in sugars, vitamins, minerals, and amino acids. The key bioactive ingredients in jujube include polysaccharides, cyclic nucleotides, polyphenols, and flavonoids, which exhibit antioxidant, immunomodulatory, antitumor, and other physiological activities (8). Jujube powder improved average daily gain (ADG), nutrient digestibility, and muscle protein content in male goats (9). It also boosted total antioxidant levels in the milk and blood of dairy goats (10).

Fermentation of jujube powder eliminates anti-nutritional factors and enhances its overall benefits compared to those of powdered jujube when used as a feed supplement for livestock (11). For instance, supplementation with fermented jujube powder (FJP) promoted a balance between intestinal microflora and the immune system in animals (12). Moreover, dietary supplementation of FJP enhanced ADG, blood antioxidant levels, and immunological protein content in bulls (13). The bacterial protein in FJP contributes to nutrient utilization in animals by stimulating the secretion of various digestive enzymes (14). These studies suggest that FJP could improve the production performance of animals attributed to its nutrient profile and antioxidant properties.

The rumen microflora is crucial for optimal nutrient absorption and overall health in ruminants (15). A previous study has demonstrated that supplementation of *Bacillus licheniformis* in the feed of Chinese Holstein cows enhances the concentration of volatile fatty acids (VFAs) in ruminal fluid (16). However, the specific effects of FJP on rumen fermentation parameters in bulls remains unclear.

Building on these previous findings, in this study, we aimed to evaluate the effects of FJP supplementation on the production performance, rumen fermentation, and serum antioxidant activity of Simmental bulls. Additionally, we identified an optimal FJP replacement strategy for intensive cattle farming. Our findings highlight the value of including FJP in the diets of bulls to improve their production performance.

## 2 Materials and methods

### 2.1 FJP preparation

Dried dates were powdered and mixed with 50% bran, 16.7% soybean meal, 30% water, and 4% (g/ton) of bacterial solution (2 × 10^8^ colony-forming units of *Candida utilis, Bacillus licheniformis*, and *Lactobacillus plantarum* at 1:1:2). Aerobic fermentation was conducted for 1 d, followed by sealed fermentation for 9 d at 37°C and drying at 65°C for 12 h. FJP comprised 89.91% dry matter (DM), 13.63% crude protein (CP), 3.38% ether extract (EE), 0.56% calcium, and 0.21% phosphorus. Additionally, the feed contained 45.82% neutral detergent fiber (NDF) and 35.99% acid detergent fiber (ADF).

### 2.2 Animals and experimental design

The study protocol was approved by the Animal Care and Use Committee of Lyuliang University and was conducted in accordance with the Guide for the Care and Use of Agricultural Animals in Research and Teaching (https://www.asas.org/services/ag-guide). Forty Simmental bulls [mean age, 311 ± 15.6 d; mean body weight (BW), 402 ± 7.2 kg] were grouped according to BW and randomly allocated to one of the four treatment groups. Bulls in the control group were fed a basal diet without FJP, whereas those in the low-, medium-, and high-dose FJP groups (LFJP, MFJP, and HFJP, respectively) were fed isoenergetic and isonitrogenous diets with 5, 7.5, and 10% FJP (DM basis), respectively. The composition and nutrient levels of the diets are shown in Table 1. The amount of FJP was determined based on a report by Zhang et al. (13). The diets were formulated based on “Nutrient Requirements of Beef Cattle: Eighth Revised Edition” (17). The experimental period was 80 d, consisting of adaptation for 20 d and a feeding trial for 60 d. The treatments were administered during the adaptation period. All bulls were housed individually, fed at 07:00 and 19:00 h daily, and had free access to feed and water.

**Table 1:** Ingredient and chemical composition of experimental diets (DM basis).

**Items**	**Contents [g/kg]**			
	**Control**	**LFJP**	**MFJP**	**HFJP**
**Ingredients**				
Corn silage	500	500	500	500
Corn grain, ground	275	242	225.5	209
Wheat bran	34	19.6	12.4	5.2
Soybean meal	120	118	117	116
Cottonseed meal	40	39.4	39.1	38.8
Fermented jujube powder	0	50	75	100
Calcium carbonate	5	5	5	5
Salt	5	5	5	5
Calcium biphosphate	15	15	15	15
Sodium bicarbonate	5	5	5	5
Mineral and vitamin premix^a^	1	1	1	1
**Chemical composition**				
NEm (MJ/kg)	7.53	7.53	7.53	7.53
NEg (MJ/kg)	5.21	5.21	5.21	5.21
Crude protein	159.1	159.1	159.1	159.1
Neutral detergent fiber	324.2	325.6	326.3	326.9
Acid detergent fiber	177.4	178.5	179.0	179.6
cAMP mg/kg	-	0.9	1.35	1.8
Polysaccharides g/kg	2.01	4.03	5.15	6.18
Polyphenols mg/kg	5.24	10.46	13.17	15.53
Flavonoids mg/kg	27.63	83.85	111.04	140.13
Calcium	6.8	6.9	7.0	7.1
Phosphorus	4.3	4.2	4.2	4.2

^a^Contained per kg premix: 100 mg Co, 8,500 mg Cu, 50,000 mg Fe, 30,000 mg Mn, 30,000 mg Zn, 300 mg I, 300 mg Se, 7,500,000 IU vitamin A, 1,200,000 IU vitamin D, and 40,000 IU vitamin E.

### 2.3 Growth performance analysis

During the feeding trial, every bull was weighed for two consecutive days before the morning diet on days 1 and 60. For each bull, the daily DM intake (DMI) was calculated as the difference between the offered and remaining feed. The feed conversion rate (FCR) was calculated as DMI/ADG.

### 2.4 Feed and feces analysis

Feed and feces were sampled on days 51–57. Fecal samples (~200 g) were collected from each bull, as described by Liu et al. (18). Each sample was dried at 65°C for 48 h and filtered through a 1-mm screen for chemical analysis. Measurements of DM (method 934.01), ash (method 942.05), CP (method 976.05), and ADF (method 973.18) were performed according to the Association of Official Analytical Chemists' guidelines (19). The amount of NDF was measured as previously described by Van Soest et al. (20). The digestibility indicator (acid-insoluble ash) was calculated as previously described by Van Van Keulen and Young (21).

### 2.5 Ruminal fluid collection and analysis

On days 58–59, the ruminal fluid of each bull was sampled, as described by Liu et al. (18). The pH of the ruminal fluid samples was measured using a pH meter (Sartorius Lab Instruments GmbH & Co. KG, Göttingen, Germany). The ruminal fluid samples were filtered through four layers of cheesecloth and the filtrates were collected. The filtrate (5 mL) was premixed with meta-phosphoric acid (1 mL; 250 g/L) and analyzed using gas chromatography (Trace 1300 gas chromatograph; Thermo Fisher Scientific, Waltham, MA, USA) with 2-ethylbutyric acid as an internal standard to estimate total VFA content of the sample (18). The filtrate (5 mL) was mixed with H_2_SO_4_ (1 mL; 20 g/L) and the ammonia nitrogen (NH_3_-N) content of the sample was estimated in accordance with the Association of Official Analytical Chemists guidelines (19). The enzyme activities of ruminal fluids were determined as described by Agarwal et al. (22).

Microbial DNA was isolated from 1 mL aliquots of blended ruminal filtrate samples as described in a previous study (23). The quality and quantity of the isolated DNA were determined using agarose gel electrophoresis and spectrophotometry (NanoDrop™ 2000 Spectrophotometer; Thermo Fisher Scientific). Microbial DNA was amplified using quantitative real-time polymerase chain reaction (RT-qPCR) carried out on a StepOne™ Real-Time PCR System (Thermo Fisher Scientific) with the primers listed in Table 2 (24). Each 20 μL RT-qPCR reaction volume included 10 μL of SYBR Green Premix Ex Taq™ II (2 × ; Takara Bio, Inc., Shiga, Japan), 2 μL of template DNA, 0.8 μL of forward and reverse primers (10 μM), 0.4 μL of ROX Reference Dye II (50 × ; Thermo Fisher Scientific), and 6 μL of ultrapure water. The PCR conditions included a denaturation step at 95°C for 2 min, followed by 45 cycles of annealing at 95°C for 15 s and elongation at 60°C for 1 min. RT-qPCR products were purified using the MiniBEST DNA Fragment Purification Kit Ver. 4.0 [Takara Biomedical Technology (Beijing) Co., Ltd., Beijing, China] and quantified using a spectrophotometer. For each standard derived from the sample, the concentration of copy numbers was determined based on the length and mass of the PCR products. Standard trajectories of specific microorganisms were charted using serial 10-fold dilutions (25). To assess amplification specificity, the dissociation patterns of the PCR end-products were determined by increasing the temperature from 60 to 95°C at 1°C/30 s.

**Table 2:** PCR primers for amplification of microbial DNA.

**Target species**	**Primer sequence (5^′^- 3^′^)**	**GenBank accession no**.	**Size (bp)**
Total bacteria	F: CGGCAACGAGCGCAACCC	AY548787.1	147
	R: CCATTGTAGCACGTGTGTAGCC		
Total fungi	F: GAGGAAGTAAAAGTCGTAACAAGGTTTC	GQ355327.1	120
	R: CAAATTCACAAAGGGTAGGATGATT		
Total protozoa	F: GCTTTCGWTGGTAGTGTATT	HM212038.1	234
	R: CTTGCCCTCYAATCGTWCT		
*Ruminococcus albus*	F: CCCTAAAAGCAGTCTTAGTTCG	CP002403.1	176
	R: CCTCCTTGCGGTTAGAACA		
*Ruminococcus flavefaciens*	F: ATTGTCCCAGTTCAGATTGC	AB849343.1	173
	R: GGCGTCCTCATTGCTGTTAG		
*Butyrivibrio fibrisolvens*	F: ACCGCATAAGCGCACGGA	HQ404372.1	65
	R: CGGGTCCATCTTGTACCGATAAAT		
*Fibrobacter succinogenes*	F: GTTCGGAATTACTGGGCGTAAA	AB275512.1	121
	R: CGCCTGCCCCTGAACTATC		
*Ruminobacter amylophilus*	F: CTGGGGAGCTGCCTGAATG	MH708240.1	102
	R: GCATCTGAATGCGACTGGTTG		
*Prevotella ruminicola*	F: GAAAGTCGGATTAATGCTCTATGTTG	LT975683.1	74
	R: CATCCTATAGCGGTAAACCTTTGG		

### 2.6 Blood collection and analysis

On day 60, blood samples were collected from each bull as described by Liu et al. (18). Total protein concentration, total antioxidant capacity (T-AOC), superoxide dismutase (SOD) activity, glutathione peroxidase (GSH-Px) activity, and malondialdehyde (MDA) content were measured using appropriate enzyme-linked immunosorbent assay kits (Thermo Fisher Scientific).

### 2.7 Calculations and statistical analysis

Trial data were analyzed using SAS software (SAS Institute, Inc., Cary, NC, USA) with a one-way analysis of variance option of the generalized linear model, and both linear and quadratic impacts were examined using orthogonal polynomials (26). A randomized block experiment format was utilized, which incorporated multiple measurements, as described by the equation Y_*ij*_ = μ + *F*_*i*_ + ε_*ij*_, where Y_*ij*_ is the dependent variable, μ is the aggregate mean, *F*_*i*_ is the constant impact of FJP addition (i = 0, 5, 7.5, and 10%), and ε_*ij*_ is the remaining error. Each bull was treated as a single unit in the experiment. Duncan's method was used for multiple comparisons, and significant differences were identified at *P* < 0.05.

## 3 Results

### 3.1 Growth performance

As shown in Table 3, dietary FJP supplementation did not affect the DMI of the bulls (*P* > 0.05). The initial and final BWs of the bulls were similar among the treatment groups (*P* > 0.05). The ADG and FCR of the bulls showed a quadratic increase (*P* = 0.049) and linear reduction (*P* = 0.042) by dietary FJP supplementation, respectively, with higher ADG (*P* = 0.035) and lower FCR (*P* = 0.033) in the MFJP group than those in the control group.

**Table 3:** Effects of fermented jujube powder (FJP) on production performance of bulls (*n* = 40).

	**Treatment** ^**a**^					***P*** **-values**		
**Item**	**Control**	**LFJP**	**MFJP**	**HFJP**	**SEM**	**Treatment**	**Linear**	**Quadratic**
DMI (kg/d)	10.70	10.72	10.81	10.73	0.100	0.069	0.196	0.078
**Body weight (kg)**								
0 d	404.4	401.6	402.0	404.1	4.179	0.329	0.933	0.070
60 d	468.6	467.4	470.5	470.2	5.978	0.640	0.364	0.816
ADG (kg/d)	1.071^b^	1.097^ab^	1.143^a^	1.101^ab^	0.057	0.035	0.076	0.049
FCR (kg/kg)	10.02^a^	9.80^ab^	9.48^b^	9.74^ab^	0.429	0.033	0.042	0.060

^a^Control, LFJP, MFJP, and HFJP groups contained respectively 0, 5, 7.5, and 10 g FJP/100 g DM. Different superscript letters in the same row differed significantly.

### 3.2 Total-tract nutrient digestibility and ruminal fermentation

As shown in Table 4, DM and CP digestibility increased quadratically with FJP supplementation (*P* = 0.026 and 0.041, respectively) and was highest in the MFJP group (*P* = 0.015 and 0.026, respectively). The total-tract apparent digestibility of organic matter and NDF were similar among the treatment groups (*P* > 0.05). ADF digestibility showed a linear increase (*P* = 0.048) with FJP supplementation and was greater in the MFJP group than that in the control group (*P* = 0.041).

**Table 4:** Effects of fermented jujube powder (FJP) on ruminal fermentation and nutrient digestibility in bulls (*n* = 40).

	**Treatment** ^**a**^					***P*** **-values**		
**Item**	**Control**	**LFJP**	**MFJP**	**HFJP**	**SEM**	**Treatment**	**Linear**	**Quadratic**
**Apparent digestibility**								
Dry matter	67.11^b^	67.42^b^	67.98^a^	67.45^b^	0.640	0.015	0.059	0.026
Organic matter	74.36	74.92	75.12	74.83	0.801	0.182	0.152	0.093
Crude protein	72.15^b^	72.39^b^	72.95^a^	72.43^b^	0.621	0.026	0.097	0.041
Neutral detergent fiber	58.72	59.02	59.64	59.42	1.169	0.309	0.108	0.481
Acid detergent fiber	53.78^b^	54.08^ab^	54.51^a^	54.16^ab^	0.583	0.041	0.048	0.068
**Ruminal fermentation**								
pH	6.46	6.44	6.43	6.42	0.046	0.373	0.090	0.786
Total VFA (mM)	116.48^b^	118.15^b^	122.77^a^	117.94^b^	5.163	0.032	0.191	0.037
**Mol/100 mol**								
Acetate (A)	66.14^b^	66.85^a^	66.93^a^	66.97^a^	0.623	0.004	0.002	0.059
Propionate (P)	20.78	20.67	20.42	20.41	0.383	0.067	0.120	0.677
Butyrate	9.34	8.86	9.24	9.09	0.548	0.233	0.634	0.348
Valerate	1.72	1.70	1.67	1.64	0.168	0.761	0.291	0.942
Isobutyrate	0.85	0.84	0.80	0.85	0.088	0.620	0.760	0.411
Isovalerate	1.19	1.08	0.94	1.04	0.213	0.081	0.057	0.118
A:P	3.18^b^	3.23^ab^	3.28^a^	3.28^a^	0.067	0.001	0.001	0.184
Ammonia N (mg/100 mL)	10.03^a^	9.85^ab^	9.65^b^	9.66^b^	0.326	0.020	0.003	0.318

^a^Control, LFJP, MFJP, and HFJP groups contained respectively 0, 5, 7.5, and 10 g FJP/100 g DM. Different superscript letters in the same row differed significantly.

Ruminal pH was similar between the treatment groups (*P* > 0.05). The rumen total VFA concentration increased quadratically (*P* = 0.037) with dietary FJP supplementation and was the highest in the MFJP group (*P* = 0.032). The acetate molar proportion showed a linear increase (*P* = 0.002) with FJP supplementation and was higher in the LFJP, MFJP, and HFJP groups than that in the control group (*P* = 0.004). FJP supplementation did not affect the molar proportions of propionate, butyrate, valerate, isobutyrate, or isovalerate (*P* > 0.05). The rumen acetate-to-propionate ratio increased linearly (*P* = 0.001) with FJP supplementation and was greater in the MFJP and HFJP groups than that in the control group (*P* = 0.001). NH_3_-N concentrations decreased linearly (*P* = 0.003) with FJP supplementation and were lower in the MFJP and HFJP groups than that in the control group (*P* = 0.020).

### 3.3 Ruminal enzyme activity and microflora

As shown in Table 5, the activities of carboxymethyl cellulase, cellobiase, pectinase, and α-amylase in the rumen were unaffected by FJP supplementation (*P* > 0.05). The activities of xylanase and protease increased linearly with dietary FJP supplementation (*P* = 0.006 and 0.018, respectively). Furthermore, xylanase activity was higher in the MFJP and HFJP groups than that in the control group (*P* = 0.031). Protease activity was also elevated in the MFJP group compared to that in the control group (*P* = 0.044). FJP supplementation quadratically increased the populations of total bacteria, *Ruminococcus albus*, and *Ruminobacter amylophilus* (*P* = 0.047, 0.011, and 0.021, respectively), and their counts were higher in the MFJP group than those in the control group (*P* = 0.026, 0.043, and 0.047, respectively). The abundance of *Ruminococcus flavefaciens* increased linearly (*P* = 0.009) and was higher in the LFJP, MFJP, and HFJP groups than that in the control group (*P* = 0.027). FJP supplementation did not affect the abundance of fungi, protozoa, *Fibrobacter succinogenes, Butyrivibrio fibrisolvens*, or *Prevotella ruminicola* (*P* > 0.05).

**Table 5:** Effects of fermented jujube powder (FJP) on rumen microbial enzyme activity and microflora in bulls (*n* = 40).

	**Treatment** ^**a**^					***P*** **-values**		
**Item**	**Control**	**LFJP**	**MFJP**	**HFJP**	**SEM**	**Treatment**	**Linear**	**Quadratic**
**Microbial enzyme activity** ^b^								
Carboxymethyl-cellulase	0.254	0.262	0.265	0.264	0.016	0.459	0.167	0.424
Cellobiase	0.166	0.170	0.178	0.174	0.013	0.194	0.081	0.342
Xylanase	0.816^b^	0.820^ab^	0.828^a^	0.828^a^	0.012	0.031	0.006	0.487
Pectinase	0.458	0.470	0.472	0.470	0.021	0.433	0.226	0.278
α-amylase	0.464	0.472	0.474	0.470	0.022	0.782	0.559	0.401
Protease	0.346^b^	0.352^ab^	0.360^a^	0.356^ab^	0.011	0.044	0.018	0.192
**Ruminal microflora (copies/mL)**								
Total bacteria, × 10^11^	3.90^b^	4.12^ab^	4.50^a^	4.18^ab^	0.461	0.026	0.051	0.047
Total anaerobic fungi, × 10^8^	3.10	3.26	3.56	3.48	0.536	0.203	0.058	0.475
Total protozoa, × 10^8^	2.14	2.36	2.35	2.25	0.278	0.242	0.407	0.067
*R. albus*, × 10^8^	5.14^b^	6.45^ab^	7.07^a^	5.78^ab^	1.637	0.043	0.244	0.011
*R. flavefaciens*, × 10^9^	3.99^b^	4.70^a^	4.93^a^	4.89^a^	0.813	0.027	0.009	0.118
*F. succinogenes*, × 10^10^	2.05	2.18	2.87	2.30	0.764	0.073	0.160	0.134
*B. fibrisolvens*, × 10^9^	4.22	4.29	4.43	4.16	1.845	0.990	0.990	0.779
*P. ruminicola*, × 10^10^	1.34	1.52	1.72	1.52	0.602	0.589	0.408	0.330
*Rb. amylophilus*, × 10^8^	1.06^b^	1.45^ab^	1.68^a^	1.34^ab^	0.510	0.047	0.115	0.021

^a^Control, LFJP, MFJP, and HFJP groups contained respectively 0, 5, 7.5, and 10 g FJP/100 g DM. Different superscript letters in the same row differed significantly.

^b^Enzymatic activity units were as follows: carboxymethyl-cellulase (μmol glucose/min/mL), cellobiase (μmol glucose/min/mL), xylanase (μmol xylose/min/mL), pectinase (μmol D-galacturonic acid/min/mL), α-amylase (μmol glucose/min/mL), and protease (μg hydrolyzed protein/min/mL).

### 3.4 Blood antioxidant properties

As shown in Table 6, the total blood protein concentration and T-AOC increased linearly (*P* = 0.003 and 0.018, respectively) with FJP supplementation. The total protein concentrations were higher in the MFJP and HFJP groups than that in the control group (*P* = 0.012). Serum T-AOC was greater in the MFJP group than that in the control group (*P* = 0.017). The activities of SOD and GSH-Px were unaffected by FJP supplementation (*P* > 0.05). The blood MDA concentration decreased linearly (*P* = 0.006) with FJP supplementation and was lower in the LFJP, MFJP, and HFJP groups than that in the control group (*P* = 0.012).

**Table 6:** Effects of fermented jujube powder (FJP) on blood antioxidant activity in bulls (*n* = 40).

	**Treatment** ^**a**^					***P*** **-values**		

**Item**	**Control**	**LFJP**	**MFJP**	**HFJP**	**SEM**	**Treatment**	**Linear**	**Quadratic**
Total protein (μg/mL)	725^b^	764^ab^	798^a^	791^a^	57.09	0.012	0.003	0.158
T-AOC (U/mL)	6.68^b^	6.75^b^	6.97^a^	6.84^ab^	0.218	0.017	0.018	0.123
SOD (U/mL)	58.86	60.49	63.20	61.22	3.603	0.209	0.087	0.250
GSH-Px (U/mL)	52.10	53.53	54.94	53.22	3.739	0.415	0.375	0.191
MDA (mmol/L)	2.36^a^	2.02^b^	1.88^b^	1.95^b^	0.366	0.012	0.006	0.055

^a^Control, LFJP, MFJP, and HFJP groups contained respectively 0, 5, 7.5, and 10 g FJP/100 g DM. Different superscript letters in the same row differed significantly.

## 4 Discussion

### 4.1 Growth performance

Dietary supplementation with FJP did not affect the DMI of bulls, which was consistent with the results of Zhang et al. (13). The observed increase in ADG could be due to improved nutrient digestibility and a higher total rumen VFA concentration. The decrease in FCR suggests that FJP supplementation enhanced the efficiency of nutrient utilization in the bulls. The positive effect of FJP on protein metabolism, indicated by an increase in total blood protein, likely contributed to the high ADG.

Cyclic adenosine monophosphate (cAMP) plays key roles in several biological processes. It alters energy partitioning in animals and energy availability is reduced by proteolysis, which subsequently increases protein deposition (27). Probiotic fermentation of jujube powder increases the concentration of cAMP in jujube (7), indicating the potency of FJP in improving the growth performance of bulls. Additionally, jujuboside, a bioactive compound found in jujube, promotes cell proliferation by regulating the cAMP-protein kinase A/cAMP-response element binding protein (PKA/CREB) signaling pathway and hormone secretion (6). Bioactive substances in jujube, including oligosaccharides and flavonoids, inhibit the proliferation of harmful bacteria, increase the abundance of probiotics in the intestine, inhibit the competition for nutrients between bacteria and the host, and improve the absorption and utilization of feed nutrients (9). A previous study on sows has demonstrated that FJP supplementation promotes protein utilization and ADG (11). However, in the present study, ADG did not increase further in the HFJP group, which is consistent with the observed nutrient digestibility and rumen VFA concentration. These findings suggest that the HFJP diet interferes with microbial growth in the rumen, resulting in decreased nutrient digestion in bulls. A previous study has shown that excessive FJP in diets result in high proliferation of *Lactobacillus* in the rumen, which could inhibit cell growth by disrupting the cell cycle (28). Moreover, Zhang et al. showed reduced ADG in Holstein dairy cattle fed 15% FJP diets compared with the control group (13). Together, these findings suggest that while FJP is beneficial in moderate amounts, excessive supplementation can be counterproductive. Therefore, careful consideration of the appropriate dosage is essential to maximize the benefits of FJP supplementation in animal diets.

### 4.2 Nutrient digestibility and ruminal fermentation

The increased digestibility of DM, CP, and ADF in the total tract was consistent with the increase in total VFA concentration and acetate molar proportion in the rumen, indicating that dietary FJP supplementation enhanced rumen nutrient degradation. A previous study on ruminants has shown that FJP increases the effective degradability of CP, EE, and ADF in the rumen (14). Furthermore, FJP alleviates digestive tract injury and promotes nutrient digestion and absorption attributed to its polysaccharide, flavonoid, and cAMP contents (13, 29). Probiotics secrete various digestive enzymes that degrade macromolecules, and the resulting metabolites enhance gastrointestinal peristalsis (30). Together, these findings indicate that dietary FJP supplementation improves the apparent nutrient digestion in the total tract. Our findings also revealed that FJP maintained rumen pH in the range of 6.42–6.46, which is conducive to microbial growth (31). Furthermore, an increase in the acetate-to-propionate ratio indicates a shift in rumen fermentation toward more acetate production. High total VFA concentrations and acetate molar proportions in the rumen are associated with increased xylanase activity, as jujube is degraded to acetate by cellulolytic enzymes (15). Similarly, dietary probiotic complexes have been found to increase rumen VFA concentrations and acetate-to-propionate ratio in sheep (32). Rumen NH_3_-N is mainly derived from feed CP degradation by rumen proteases (33). However, the decrease in rumen NH_3_-N concentration by FJP supplementation was inconsistent with the observed increase in protease activity, which could be associated with increased microbial protein content, as cellulolytic bacteria utilize NH_3_-N for protein synthesis (34). These findings collectively indicate that FJP supplementation positively influences rumen fermentation and nutrient digestibility, leading to better nutrient absorption and utilization in bulls.

### 4.3 Ruminal enzyme activity and microflora

The observed increase in xylanase activity coincided with an increase in the population of cellulolytic bacteria, indicating that FJP supplementation stimulated the secretion of enzymes by microbes. Cellulolytic bacteria produce enzymes that degrade fibers (15). The increase in protease activity is associated with an increase in the proportion of *Ruminobacter amylophilus*, which is responsible for protein degradation (35). These results suggest that dietary FJP supplementation promotes the growth of rumen microorganisms and enzyme secretion in bulls. Additionally, cAMP promotes cell proliferation via the PKA/CREB signaling pathway (6). FJP supplementation has been reported to promote the colonization of probiotics in the gastrointestinal environments and regulate the balance of microflora through biological oxygen capture and competitive inhibition (36). Probiotics can prevent the colonization of pathogens by niche pre-emption (37). Dietary supplementation with probiotic complexes has been found to increase the abundance of *Ruminococcus albus, F. succinogenes, Butyrivibrio fibrisolven*s, and *P. ruminicola* in the rumen of bulls (30). Consistently, a previous study has shown that probiotic supplementation increases the proportions of *Butyrivibrio fibrisolvens* and *Ruminococcus flavefaciens* in the rumen of sheep at a dietary concentrate-to-roughage ratio of 3:7 (38). Together, these findings suggest that FJP serves as a valuable dietary supplement to improve rumen microbes and rumen fermentation in livestock.

### 4.4 Blood antioxidant properties

Oxidative stress in farming bulls is a major concern because it negatively affects performance and meat quality and is associated with high morbidity. In this study, serum T-AOC significantly increased with FJP supplementation, consistent with the results of Zhang et al. (13). Additionally, Xu et al. (7) reported that the hydroxyl groups of polyphenols in jujube can chelate Cu^2+^ and Fe^2+^ to reduce oxidation rates, whereas the phenolic hydroxyl groups of flavonoids form stable semiquinone free radical structures, reducing free radical accumulation. The high polyphenol content in FJP is attributed to the release of bound phenols from the cell wall, which are degraded by cellulases secreted by *Bacillus* spp. and *Lactobacillus plantarum* (39). These findings suggest that an increase in T-AOC due to FJP supplementation may conserve the protein content and improve the production performance of bulls.

## 5 Conclusion

In conclusion, this study demonstrates that dietary supplementation with FJP has beneficial effects on multiple aspects of production performance, rumen fermentation, and antioxidant properties in Simmental bulls. Our findings indicate that FJP enhances ADG and improves feed conversion efficiency, suggesting improved nutrient utilization and growth. The observed changes in rumen fermentation, including increases in total VFA and acetate molar proportion, along with reduced NH3-N concentrations, highlight the role of FJP in optimizing digestive processes and nutrient metabolism. Furthermore, the significant improvements in blood T-AOC and reduction in MDA levels indicate enhanced antioxidant defenses, potentially reducing oxidative stress in supplemented bulls. These results underscore the potential of FJP as a valuable dietary supplement for enhancing growth performance in cattle farming practices. Future research should further explore optimal dosages and long-term effects of FJP supplementation across different cattle breeds and production environments to fully leverage its benefits in livestock management strategies.

## Data availability statement

The original contributions presented in the study are included in the article/Supplementary material, further inquiries can be directed to the corresponding author.

## Ethics statement

The animal study was approved by the Animal Care and Use Committee of Lyuliang University. The study was conducted in accordance with the local legislation and institutional requirements.

## Author contributions

YL: Conceptualization, Data curation, Formal analysis, Funding acquisition, Methodology, Software, Validation, Writing – original draft, Writing – review & editing. GW: Conceptualization, Supervision, Validation, Writing – review & editing. RW: Conceptualization, Data curation, Validation, Writing – original draft. XZ: Conceptualization, Validation, Writing – original draft. CF: Project administration, Resources, Writing – review & editing. YH: Investigation, Writing – original draft. PC: Formal analysis, Investigation, Supervision, Writing – review & editing.
